# Treatment confidence and patient participation in multidisciplinary tumor conferences: A structural equation modeling approach

**DOI:** 10.1002/cam4.7199

**Published:** 2024-05-27

**Authors:** Marie Degenhardt, Nicole Ernstmann, Barbara Schellenberger, Lena Ansmann, Christian Heuser

**Affiliations:** ^1^ Department for Psychosomatic Medicine and Psychotherapy, Faculty of Medicine, Center for Health Communication and Health Services Research University Hospital Bonn Bonn Germany; ^2^ Center for Integrated Oncology Aachen Bonn Cologne Düsseldorf (CIO ABCD) Bonn Germany; ^3^ University of Cologne, Faculty of Medicine and University Hospital Cologne, Institute of Medical Sociology, Health Services Research and Rehabilitation Science, Chair of Health Services Research Cologne Germany; ^4^ University of Cologne, Faculty of Medicine and University Hospital Cologne, Institute of Medical Sociology, Health Services Research and Rehabilitation Science, Chair of Medical Sociology Cologne Germany

**Keywords:** breast cancer, decision‐making, integrative model of patient‐centeredness, multidisciplinary tumor conferences, patient participation, patient‐centered care, psycho–oncology, structural equation modeling analysis

## Abstract

**Objective:**

Multidisciplinary tumor conference (MTC) is a key instrument in multidisciplinary cancer care. In recent years, if and how patient participation in MTC can contribute to a more patient‐centered care have been scientifically discussed. This study aimed to identify determinants of treatment confidence in the context of patient participation in MTC. Therefore, the association among health literacy‐sensitive communication, trust in health‐care providers (HCP), and treatment confidence is examined.

**Methods:**

This study used data from the multicenter, observational study “PINTU” on patient participation in MTC. Data were collected from November 2018 to February 2020. Validated scales for treatment confidence, health literacy‐sensitive communication, and trust in providers were included in the structural equation modeling (SEM) analysis.

**Results:**

A total of 95 patients participated in MTC. The sample compromised *n* = 80 completed datasets. The SEM fit measures indicated good fit of the proposed model. The analysis showed a positive association between health literacy‐sensitive communication and treatment confidence when adding the mediating effect of trust in providers.

**Conclusion:**

Patient‐centered communication during MTC in combination with a trustful relationship between participating patients and health‐care providers is positively associated with treatment confidence. The results indicated the relevance of a trustful doctor–patient communication and relationship. Trainings for physicians targeting patient‐centered communication could be a promising approach to strengthen patient participation.

## 1 BACKGROUND

Cancer treatment involves a complex set of decisions that must meet both individualized care and guideline adherence.^1^ Guidelines offer consensus‐ and evidence‐based recommendations for health‐care providers (HCP) and patients,^2^ such as the German *interdisciplinary S3 guideline for the early detection, diagnosis, therapy, and follow‐up of breast carcinoma* of the AWMF (Arbeitsgemeinschaft der Wissenschaftlich‐Medizinischen Fachgesellschaft). The quality of guidelines in the oncological field is particularly high.^3^ However, HCP report encountering challenges in applying the guidelines in practice.^4^ These challenges may arise because guidelines are based on simplified and specific conditions and do not consider factors such as patients' preferences, personal circumstances, or accompanying illnesses and comorbidities specific to the tumor. To address this complexity, multidisciplinary tumor conferences (MTCs) are a guideline‐based element in the decision‐making process that are expected to weigh treatment options, support clinical decision‐making, and improve patient outcomes.^5^ MTCs are routinely held meetings of a multidisciplinary team of HCP coming together to discuss the diagnoses as well as an individualized and optimized treatment plan for each patient.^6^ Results from an umbrella review indicate that MTCs have a positive effect on diagnostic assessment and staging accuracy, treatment management, guideline adherence, and health outcomes.^7^ So far, the focus of the multidisciplinary discussion has been clearly on medical aspects, whereas patients' perspective, psychosocial background, and comorbidities have received less attention.^8^, ^9^ However, not considering patients' wishes, preferences, life circumstances, and comorbidities negatively affects the actual implementation of these recommendations. Previous studies have demonstrated that between 36% and 65% of recommendations are not implemented after an MTC due to the lack of knowledge about patients' preference.^10^, ^11^

The patient‐centered approach calls for attention to and inclusion of patient‐specific information and can therefore contribute to patient empowerment.^12^ One of the prevailing theoretical foundations is the integrative model of patient centeredness by Scholl et al.^13^ This model is based on three components: (i) principles (subcategories: essential characteristics of the clinician, clinician–patient relationship, recognition of the patient as a unique person, and biopsychosocial perspective), (ii) enablers (subcategories: clinician–patient communication, integration of medical and nonmedical care, teamwork and teambuilding, patients' access to care, and coordination and continuity of care), and (iii) activities (subcategories: patient information, patient involvement in care, patient empowerment, and emotional and physical support).

In recent years, MTCs have been increasingly questioned scientifically as regards their contribution to patient‐centered care.^8^ One way to potentially ensure patient‐centeredness in MTC might be through the participation of the patients themselves.^14^ Heuser et al. identified 6.9% participating patients in a sample of 863 patients from 43 randomly selected German breast cancer centers.^15^ So far, an invitation by the treating physicians has only been given in rare cases, but when offered, it is accepted in about half of the cases.^16^ Patients report both their positive and negative experiences, with up to 90% not regretting the participation or perceiving it as helpful.^17^ The predictors of participation were hospital‐related criteria and higher health literacy.^15^

Higher health literacy is associated with better patient‐reported outcomes (PRO), patient empowerment, and patient preference for patient‐centered care.^18^ Health literacy‐sensitive communication is defined as physicians' ability to respond to patients' level of health literacy and can be seen as part of organizational health literacy.^19^ As a construct of patient‐centered communication, which is rarely observed in MTC,^20^ health literacy‐sensitive communication could be a crucial factor for the effects of patient participation in MTC as it also has an impact on patient self‐reported empowerment, moderated by the perceived support by physicians.^19^ Higher health literacy‐sensitive communication was predictive for fewer unmet information needs and higher levels of perceived support from physicians.^21^ Increased trust in HCP, which also plays a crucial role in patient–physician relationship and is one of the most important factors in the decision‐making process for patients,^22^ was associated with positive changes in physical functioning and patient empowerment.^23^ Health literacy‐sensitive communication and trust seem to be predictive patient‐reported experiences (PREMs), which can contribute to a more patient‐centered and effective care^24^; however, little is known about their association in the context of patient participation in MTC. Treatment confidence appears to be an underutilized but promising PRO in this regard, as it had a positive effect on beliefs about the manageability and usefulness of treatment and on an uncomplicated course of recovery.^25^ Owing to the heterogeneous retrospective evaluation of patient participation in MTC, it is unclear which factors cause them to feel confident about their treatment and the progression of disease.^16^

**FIGURE 1:**
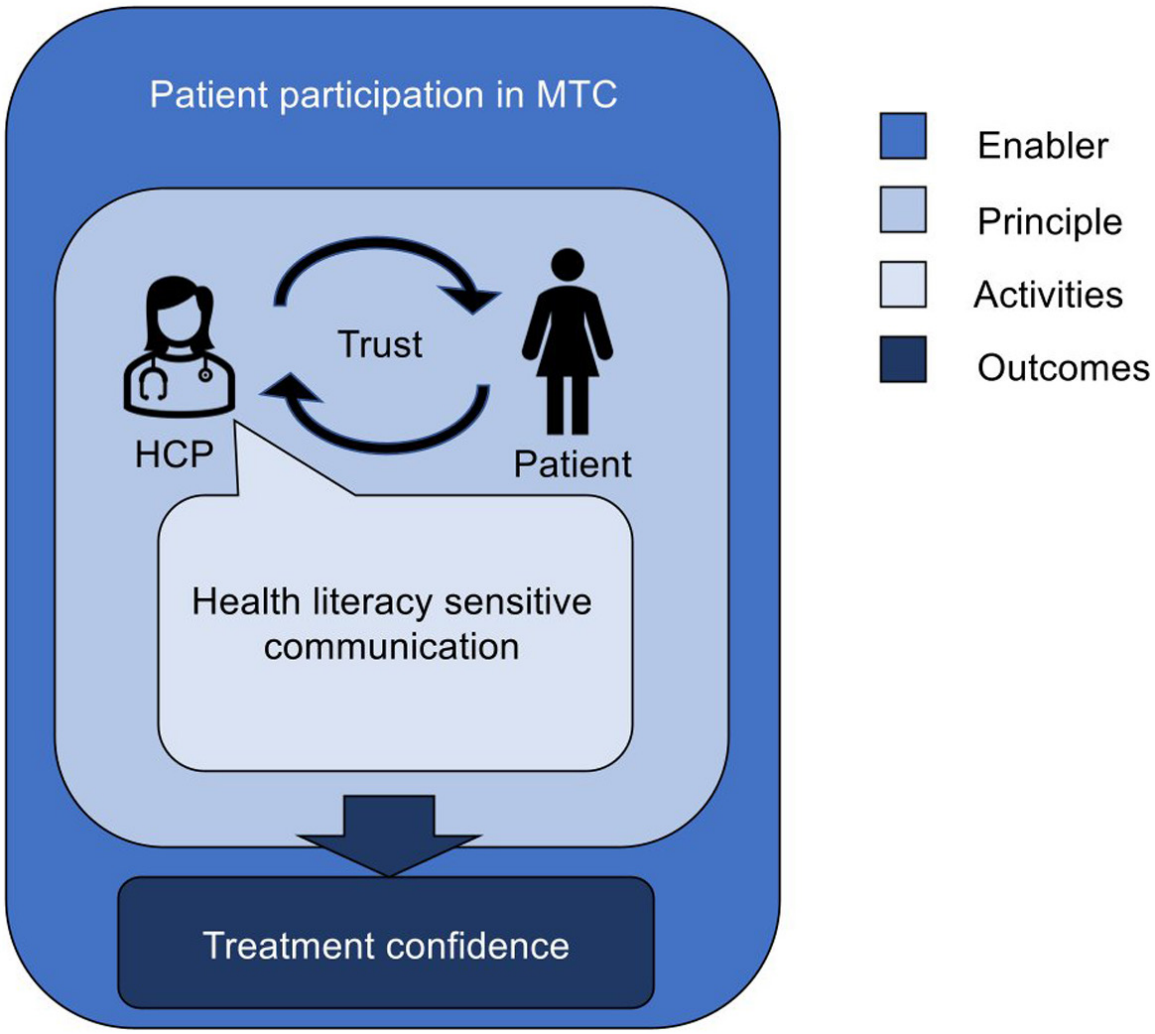
Embedded constructs of health literacy‐sensitive communication, trust in providers, and treatment confidence in the integrative model of patient‐centeredness by Scholl et al.^13^

Placement of these constructs in the integrative model of patient‐centeredness provides evidence for their interaction.^13^ Here, as presented in Figure 1, health literacy‐sensitive communication is assigned to the category activities and trust to the dimension of patient–provider communication (category principles). This relationship is framed by the context of patient participation in MTC, which is represented by the category of enablers in the model.

Patient participation in MTC can therefore be seen as a patient‐centered care process, but it is a poorly studied phenomenon. The effects on PROs, such as treatment confidence, have not received attention, to the authors' knowledge. Therefore, this study aimed to identify the association among health literacy‐sensitive communication, trust in treatment providers, and treatment confidence. The hypotheses are as follows:

It is assumed that a high level of health literacy‐sensitive communication is positively associated with trust in providers and treatment confidence.

More concretely, it is assumed that exogenous variable health literacy‐sensitive communication is positively associated with endogenous variable treatment confidence, mediated by trust in providers.

## 2 METHODS

### 2.1 Study design and sample

PINTU (Patient Participation in Multidisciplinary Tumor Conferences in Breast Cancer Care: An exploratory study, 2017–2020) was a multicenter, observational mixed‐method study.^26^ It was funded by the German Cancer Aid (Deutsche Krebshilfe e.V.) and received a positive ethics in April 2018 from the Ethics Committee of the Medical Faculty of the University of Cologne (17–405). The PINTU study aimed to examine the feasibility of patient participation in MTC and to record the experiences of those involved. Between November 2018 and February 2020, qualitative and quantitative data were collected from patients and HCP as well as by observation from the MTCs in a total of six breast cancer centers in North Rhine‐Westphalia, chosen purposeful sampling‐wise. Three breast cancer centers participated in which patient participation in MTC was already implemented, whereas the other three breast cancer centers did not invite patients to MTC. The sampling criteria for selecting the breast centers included size (based on case volume), teaching status, and geographical location (rural and urban). Qualitative data were collected through semistructured interviews with the HCP and passive participant observations at the MTCs with and without patient participation. Quantitative data were collected using standardized, paper‐based patient questionnaires before (T0) and 4 weeks after the MTC (T2). The questionnaires were tested for cognitive strain in a pretest phase using the think‐aloud method with a comparable group of patients from self‐help groups. Patients who participated in the MTC filled out a questionnaire directly after MTC participation (T1). After the MTC and the distribution of the questionnaires by the research team, patients were offered the opportunity to talk to the responsible nurse or doctor in order to clarify questions or seek mental support. The questionnaire was returned by post by the patients. Participants received two personalized reminders according to the total design method of Dillman in order to minimize the dropout rate.^27^ The response rate was 92% for T0, 86% for T1, and 85% for T2. The following inclusion criteria for study participation were applied: age ≥18 years; diagnosis of breast cancer or other gynecological cancer (ICD 10 C50.xx – C58.xx, D05.xx – D07.xx); sufficient German language skills; sufficient physical, psychological, and cognitive abilities to complete the consent form and questionnaires; and signed informed consent form to participate in the study.

Further information about the procedure of the PINTU study is given elsewhere.^26^

### 2.2 Instruments

Health literacy‐sensitive communication was measured using the HL‐COM scale.^19^ It comprises a 4‐point Likert scale (1 = strongly agree, 2 = tend to agree, 3 = tend to disagree, and 4 = disagree at all) with a total of nine items. The questions relate, for example, to the perceived conveyance of information by the physician and its comprehensibility. The scale was inverted to be comparable to the other two scales in a sense that higher values indicate a stronger expression of the construct. For HL‐COM, this means higher values indicate a more health literacy‐sensitive communication.

Treatment confidence was measured using a single item, which was taken and adapted from the Cologne Patient Questionnaire for Breast Cancer 2.0 (KPF‐BK 2.0).^28^ The item was rated on a 10‐point Likert scale (1 = not confident to 10 = confident). The exact wording of the item is “How confident are you that the therapy (surgery, chemotherapy, pain management) you received at your cancer center is effective?”. Validations with a comparable group of female breast cancer patients are available for both the HL‐COM scale and the KPF‐BK.^19^, ^29^

Trust in providers was measured using the scale “Trust in physicians” from the Cologne Patient Questionnaire (KPF) and adapted with regard to the term “treatment team.”^29^ All five items of the scale were measured on a 6‐point Likert scale (1 = never, 2 = rarely, 3 = sometimes, 4 = often, 5 = very often, and 6 = always) and included, for example, questions regarding the perceived competence and openness of the treatment team. Relative sum score was calculated for both scales.

### 2.3 Data analysis

Descriptive data analysis was conducted using IBM SPSS version 27. Cronbach's alpha was calculated for each of the scales. Examination of hypothesis was realized using a structural equation model (SEM) with the program IBM AMOS version 27.^30^ SEM offer the possibility of validly mapping latent constructs using multiple indicator variables, as well as examining mediator variables and thus indirect effects. In the reflective model, health literacy‐sensitive communication was used as the exogenous variable and trust in providers (mediating effect) and treatment confidence as the endogenous variables. Normality testing was conducted using the skewness and kurtosis indices of the variables and compared with the critical indices according to West et al. (skewness < |2|; kurtosis < |7|).^31^ The chi‐square (*χ* ^2^) test and the common inferential statistical measures according to Hair et al. were calculated. For comparison of the default and independence model, the Tucker–Lewis index (TLI) and comparative fit index (CFI) were used, which are both relatively robust against a violation of the normal distribution and can also be applied to small samples.

Three models were compared for which (1) error variants were not allowed, (2) according to the validation article, or (3) according to the modification indices (of model 1) allowed. To test the hypotheses, the model with the best fit was selected and tested using the critical ratios, squared multiple correlations, and total effects according to the critical values of Hair et al.^30^ Datasets with missing values were excluded listwise from the analysis.

## 3 RESULTS

### 3.1 Descriptive analysis

For descriptive statistics, data are displayed for all (*n* = 95) participating patients and patients included in the SEM analysis (*n* = 80). The average age of the patients was 59 years old, and most suffered from initial cancer disease (77%/85%). Only 5.3%/6.3% suffered from metastases, whereas at the same time, 26.3%/28.7% stated that lymph nodes were affected. A total of 41.1%/43.8% of patients have a university degree and about half (48.4%/48.8%) were employed at the time of completing the questionnaire. All demographic and clinical information are presented in Table 1.

**TABLE 1:** Participants' characteristics (for all participating patients [*n* = 95] and subpopulation used for SEM [*n* = 80]).

Variables	Categories	Participating patients	Patient data used for SEM
Age (years)	*n*	*n* = 87	*n* = 77
	Mean (SD)	59.14 (10.9)	59.20 (10.9)
	Median	58	58
Family status (%)	Married	53 (55.8%)	50 (62.5%)
	Divorced	11 (11.6%)	9 (11.3%)
	Widowed	9 (9.5%)	6 (7.5%)
	Single	13 (13.7%)	11 (13.8%)
	Missing	9 (9.5%)	4 (5%)
Having children (%)	Yes	67 (70.5%)	60 (75%)
	No	20 (21.1%)	17 (21.3%)
	Missing	9 (9.5%)	3 (3.8%)
Highest level of school education (%)	No lower secondary school education	1 (1.1%)	0 (0%)
	Lower secondary school education	21 (22.1%)	20 (25%)
	Intermediate secondary school education	24 (25.3%)	20 (25%)
	University entrance certificate	39 (41.1%)	35 (43.8%)
	Other	1 (1.1%)	1 (1.3%)
	Missing	9 (9.5%)	4 (5%)
Currently employed (%)	Yes	25 (26.3%)	23 (28.7%)
	No	46 (48.4%)	39 (48.8%)
	Missing	24 (25.3%)	18 (22.5%)
Distress thermometer^a^ (T2)	*n*	*n* = 68	*n* = 64
	Mean (SD)	5.19 (2.62)	5.14 (2.66)
	Median	5	5
Cancer frequency (%)	Initial	73 (76.8%)	86 (85%)
	Repeated	12 (12.6%)	12 (15%)
Lymph node infestation (%)	Yes	25 (26.3%)	23 (28.7%)
	No	56 (58.9%)	53 (66.3%)
	Don't know	3 (3.2%)	3 (3.8%)
	Missing	11 (11.6%)	1 (1.3%)
Metastasis (%)	Yes	5 (5.3%)	5 (6.3%)
	No	71 (74.7%)	67 (83.8%)
	Don't know	8 (8.4%)	7 (8.8%)
	Missing	11 (11.6%)	1 (1.3%)
Other diseases such as hypertension, diabetes, and mental disorder (%)	Yes	39 (41.1%)	38 (47.5%)
	No	44 (46.3%)	40 (50%)
	Don't know	1 (1.1%)	1 (1.3%)
	Missing	11 (11.6%)	1 (1.3%)

^a^ Scale from 0 = not distressed at all to 10 = extremely distressed.

### 3.2 Structural equation modeling: Determinants of treatment confidence

**FIGURE 2:**
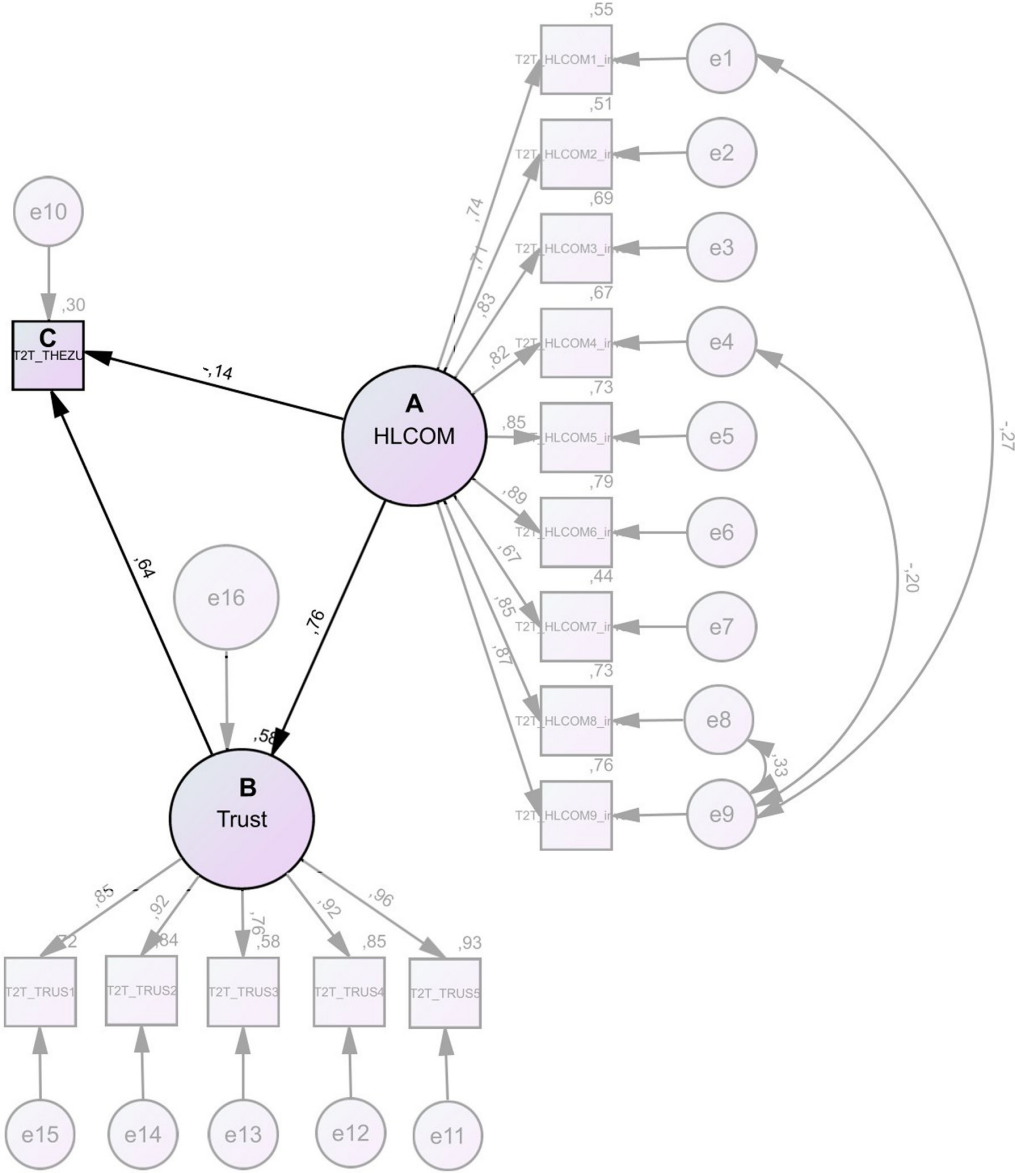
SEM with (A) health literacy‐sensitive communication (HL‐COM), (B) trust in providers (Trust), and (C) treatment confidence (T2T_THEZU) as latent variables. Direct effects are shown on errors between A, B, and C.

For the SEM, items of the three variables (health literacy‐sensitive communication, trust in providers, and treatment confidence) were entered into the proposed model. Negative skewness indices, which did not exceed the critical value of |2| for all but one variable, indicated a left‐skewed distribution (see Appendix, Tables A1 and A2).^31^ All kurtosis indices remained below the critical value of |7|. Although the available data deviated from the normal distribution, they resembled it to the extent necessary for the SEM calculation. Cronbach's alpha values were 0.946 for trust in providers and 0.942 for the inverted health literacy‐sensitive communication scale. A path diagram is presented in Figure 2. With 85° of freedom, the identifiability of the model is given. Model fit parameters are presented in Table 2. For model fit reasons, the model that allowed covariation between errors according to the modification indices of the model without covariation was chosen. The chi‐square (*χ* ^2^) value has a nonsignificant *p*‐value of 0.058 and thus does not lead to rejection of the model. As can be seen in Table 2, all fit measures indicate a good fit of the (final) model. An exploratory factor analysis was conducted, which confirmed the factor structure described in the validation articles.

**TABLE 2:** Observed and critical fit indices of the final SEM.

Model fit parameters	Observed values (SEM)	Critical values
*χ* ^2^	106.356	
*p*‐value	0.058	*p* < 0.05
Df	85	
RMSEA	0.056	≥ 0.05 = good fit
CMIN/DF	1.251	< 2.0
SRMR	0.0418	≤ 0.10 = good fit
TLI	0.975	≥ 0.90 = good fit
CFI	0.980	≥ 0.90 = good fit

Abbreviations: CFI, comparative fit index; CMIN/DF, *χ* ^2^‐value/df; Df, degrees of freedom; RMSEA, root mean square index; SRMR, standardized root mean square residual (Bentler, 2006); TLI, Tucker–Lewis index; χ2, Chi‐square.

Critical ratios of the unstandardized regression weights (see Appendix, Tables A1 and A2) indicate whether the parameter has a significant effect on the model, and path coefficients indicate the strength of that effect. All but one are significant and have a relevant correlation strength of >0.20 as seen in the path coefficients. The squared multiple correlations (Table 3) provide information on the variance explanation of the variable treatment confidence by the variables health literacy‐sensitive communication and trust in providers. Table 3 shows that five variables have a substantial effect (>0.75) according to Hair et al., nine have a moderate effect (>0.50), and the remaining two have a weak effect (>0.25).^30^ The standardized total effects (Table 3) in combination with the direct effects are presented in Figure 2, which are essential for hypothesis testing. The direct effect shows the correlation of health literacy‐sensitive communication with trust in providers (0.76) and with treatment confidence (−0.14). The standardized total effect takes into account the variable trust in providers as a mediator variable in the relationship between health literacy‐sensitive communication and treatment confidence. Thus, the effect of health literacy‐sensitive communication on treatment confidence changes to 0.35 with the addition of the mediator trust (Table 3).

**TABLE 3:** Squared multiple correlation and standardized total effects^a^.

Variables / Scales	Squared multiple correlation	Standardized total effect HL‐COM	Standardized total effect Trust
Trust	0.580	0.761	0.000
T2T_TRUS1	0.717	0.645	0.847
T2T_TRUS2	0.841	0.698	0.917
T2T_TRUS3	0.577	0.578	0.760
T2T_TRUS4	0.853	0.703	0.924
T2T_TRUS5	0.930	0.734	0.964
T2T_THEZU	0.296	**0.350** ^***^	0.641
T2T_HLCOM9_invers	0.763	0.874	0.000
T2T_HLCOM8_invers	0.728	0.853	0.000
T2T_HLCOM7_invers	0.444	0.667	0.000
T2T_HLCOM6_invers	0.793	0.890	0.000
T2T_HLCOM5_invers	0.729	0.854	0.000
T2T_HLCOM4_invers	0.668	0.818	0.000
T2T_HLCOM3_invers	0.690	0.831	0.000
T2T_HLCOM2_invers	0.508	0.713	0.000
T2T_HLCOM1_invers	0.549	0.741	0.000

^***^ *p*‐value <0.001.

^a^ According to Hair et al., SMC >0.25 = weak effect, >0.50 = moderate effect, and >0.75 = substantial effect; Variable codes: T2, timepoint; T, Teilnahme (=participation);TRUS, scale “trust in HCP”; HL‐COM_invers, inverted scale “health literacy‐sensitive communication”; THEZU, scale “treatment confidence”.

## 4 DISCUSSION

This study aimed to identify the determinants of treatment confidence in the context of patient participation in MTC. Therefore, the extent to which health literacy‐sensitive communication, trust in providers, and treatment confidence are interrelated is examined. For this purpose, a SEM was proposed, which assumed that health literacy‐sensitive communication is positively associated with trust in providers and treatment confidence (Hypothesis 1A). Furthermore, this association was expected to be mediated by trust in providers (Hypothesis 1B). The model was fundamentally confirmed and exhibits a good fit to the available data of *n* = 80 patients participating in MTC.

Hypothesis 1A is confirmed only partially. The positive association between health literacy‐sensitive communication and trust in providers could be demonstrated. This correlation is consistent with the results of studies showing the effects of physician communication on patient trust.^32^ Contrarily, the effect of the latent variable health literacy‐sensitive communication on treatment confidence (as seen in the direct effect, as well as in the critical ratios and path coefficients) was not significant but, contrary to expectations, negative. This result seems surprising as health literacy‐sensitive communication has exerted somewhat positive effects on patient empowerment,^19^ which in turn was positively associated with treatment confidence.^33^ However, the particular context of patient participation in MTC might explain this disagreement. It has been discussed that MTC participation can have a negative effect on patients and may lead to increased distress due to information overload and mental strain.^34^ Another explanatory approach is provided by the conceptual model of the oncologist–patient relational communication involvement (RCI).^35^ According to the RCI model, health literacy‐sensitive communication as purely instrumental communication without a relational component can have a negative effect on health outcomes, such as treatment confidence. In MTC, mainly instrumental communication can be observed,^9^, ^20^ and the item formulation of the HL‐COM scale focuses on patients' understanding of the medical–technical information.

The results support the acceptance of Hypothesis 1B and thus the mediating effect of trust in providers in the association between health literacy‐sensitive communication and treatment confidence. Trust is already known to determine the correlation between patient–provider communication and health outcomes.^36^ These findings highlight the importance of a good doctor–patient relationship and the fact that health literacy‐sensitive communication has positive effects on treatment confidence only under the premise of a trustful relationship.

### 4.1 Study strengths and limitations

Due to purposeful sampling of the participating centers, the sample of female breast cancer patients in this study might be selective and limit generalizability. Nevertheless, within the centers, which offer the possibility of patient participation in MTC, all patients were invited to participate in this study. The descriptive analysis of the sample indicates that it is comparable to the average of breast cancer patients in Germany. However, the high proportion of academics suggests an above‐average level of education. This, along with their interest in participating in this study, may indicate a selection bias in the inclusion process. Multivariate normal distribution is a basic prerequisite for SEM calculation.^30^ Due to the Likert scales used and thus the questionable interval scaling of the data, a normal distribution is not given in most cases of research in human and social sciences. Thus, the data in this study also do not meet the strict criteria of normal distribution; but, according to West et al., this study has skewness and kurtosis indices to calculate and interpret a SEM.^31^ Model estimation using the maximum likelihood method may result in an overestimation of the model fit in cases of high multivariate kurtosis.^37^ Due to the correlative approach, the direction of the associations must be inferred from the existing literature, whereby some correlations, such as that between HL‐COM and trust in providers (−0.14), must be considered as weak. Due to the observational and therefore noninterventional exploratory study design and the latent constructs of communication and trust, which are difficult to manipulate empirically, the results of this study can only be interpreted correlatively and not causally. Furthermore, descriptive characteristics such as lymphatic system invasion and metastasis were not included in the structural equation model, thus neglecting potential influences particularly on treatment confidence.

The strengths of this study are the established approach of SEM, for which the variables and scales are well suited and which is based on the theoretical model of patient‐centeredness by Scholl et al.^13^ To the best of the authors' knowledge, this is the first study to confirm that, in the context of patient participation in MTC, health literacy‐sensitive communication is associated with patient treatment confidence when a trustful relationship has been established. Due to the rarity of patient participation in MTC, the sample of *n* = 95 women is relatively large.

### 4.2 Clinical implications

The promotion of health literacy‐sensitive communication for physicians can be identified as a possible field of education and training. This is supported by the (American) National Action Plan to Improve Health Literacy, which advocates that the responsibility to improve health literacy lies not only with patients but also with physicians and should be addressed individually by physicians according to patients' competence.^38^ Health‐care organizations, such as cancer centers, should take concrete and practical steps in sense of patient‐centered care to bridge the gap between patients' health literacy and the complexity of care processes or facilities.^39^ Promising approaches have already been established to promote health literacy‐sensitive communication among medical students.^40^ However, to ensure that physicians are capable of establishing a trusting relationship with patients in addition to instrumental communication, the patient‐centered approach to care must be considered in the larger context of oncological care. Evidence‐based and patient‐centered approaches are often perceived as competing constructs.^41^ In this context, MTCs are considered a core element of evidence‐based medicine.^42^ Ansmann and Pfaff describe clinical guidelines as a prototype for standardization (as opposed to individualization) in the health‐care system.^43^ However, MTCs could act as a potential bridge between individualization and standardization (“individualized standardization”) by providing a guideline orientation, simultaneously focusing on the biological, psychological, social, and cultural dimensions as well as patients' wishes, needs, and preferences.^43^ The PINTU study and the here presented results discussed the extent to which a bridge can be built between evidence‐based and patient‐centered care via patient participation in MTC. Concerning patient‐centered care, the results indicate that the instrumental and relational communication behaviors of physicians themselves contribute to the treatment confidence of participating patients. The communication behavior of physicians is associated with PRO in cancer patients and may contribute to the positive effects of participation. It is an empirical example of the patient‐centered care approach. Discussions preceding the MTC to satisfy the need for information and the perceived participation of patients in decision‐making have already proven useful.^44^ Furthermore, specific training in patient‐centeredness for HCP could be offered.^45^ Especially for patients with low health literacy, patient advocates such as nurses could represent patient perspective in MTC and contribute to increased patient‐centeredness.

## 5 CONCLUSION

MTCs are a core element in the care process for patients with cancer, contribute to increased guideline orientation and improvements in diagnosis and treatment planning, and ultimately lead to better clinical outcomes for patients; however, it still lacks patient‐centeredness to reach their full potential. The inclusion of patient‐centered information, as well as patients themselves, could lead to MTC recommendations that are more likely to be implemented and contribute to patient empowerment. The study results indicate that the experiences of patients participating in MTC, represented by treatment confidence in this analysis, are dependent on physician communication behavior and a trustful relationship. Treatment confidence could be potentially increased by a trustful relationship and by combining it with health literacy‐sensitive communication. An improvement of both, for example, through trainings for treatment providers, is therefore desirable and should be further empirically investigated for feasibility and benefit. In addition, further research is needed regarding the feasibility, efficacy, effectiveness, and potential target groups of patient participation in MTC. Thereby, it is important to identify the determinants that influence success within RCT studies. There is no fundamental contradiction between evidence‐based and patient‐centered medicine, and the results of this study speak for the possibility of integrating both constructs in everyday clinical practice. MTC can be a care process in which a promising bridge is built between the patient's disease and the legitimate interest in the person behind the disease.

## AUTHOR CONTRIBUTIONS

**Marie Degenhardt:** Conceptualization (equal); data curation (equal); formal analysis (lead); methodology (equal); visualization (lead); writing – original draft (lead); writing – review and editing (lead). **Christian Heuser:** Conceptualization (equal); data curation (equal); formal analysis (equal); methodology (equal); project administration (equal); supervision (equal); validation (equal); visualization (supporting); writing – review and editing (equal). **Nicole Ernstmann:** Conceptualization (equal); data curation (supporting); formal analysis (supporting); funding acquisition (lead); methodology (equal); project administration (equal); supervision (equal); validation (equal); writing – review and editing (equal). **Barbara Schellenberger:** Data curation (equal); project administration (equal); writing – review and editing (equal). **Lena Ansmann:** Conceptualization (equal); data curation (equal); funding acquisition (lead); project administration (equal); validation (equal); writing – review and editing (equal).

## Data Availability

Data for this study are kept at the Center for Health Communication and Health Services Research, University Hospital Bonn, University of Bonn, Germany. The datasets generated and analyzed during this study are not publicly available due to terms of written informed consent to which the participants agreed but are available from the corresponding author on reasonable request.

## APPENDIX A

**TABLE A1:** Skewness, kurtosis, and Cronbach's alpha of scales.

	Skewness	Kurtosis	Cronbach's alpha
Trust			0.946
T2T_TRUS1	−1.330	0.620	
T2T_TRUS2	−1.870	2.691	
T2T_TRUS3	−1.793	3.097	
T2T_TRUS4	−1.748	2.821	
T2T_TRUS5	−2.106	4.145	
T2T_THEZU	−1.212	0.898	
HLCOM_invers			0.942
T2T_HLCOM9_invers	−1.199	1.148	
T2T_HLCOM8_invers	−1.302	1.130	
T2T_HLCOM7_invers	−0.977	0.242	
T2T_HLCOM6_invers	−0.782	−0.411	
T2T_HLCOM5_invers	−1.085	0.344	
T2T_HLCOM4_invers	−1.065	0.551	
T2T_HLCOM3_invers	−1.411	1.180	
T2T_HLCOM2_invers	−1.186	1.609	
T2T_HLCOM1_invers	−1.206	1.132	

*Note*: Variable codes: T2, timepoint; T, Teilnahme (=participation); TRUS, scale “trust in HCP”; HL‐COM_invers, inverted scale “health literacy‐sensitive communication.”

**TABLE A2:** Critical ratios of the unstandardized regression weights and path coefficients (standardized regression weights).

	Critical ratio	Standardized regression weights
Trust←HLCOM	6.677***	0.761
T2T_HLCOM1_invers←HLCOM	—	0.741
T2T_HLCOM2_invers←HLCOM	6.453***	0.713
T2T_HLCOM3_invers←HLCOM	7.606***	0.831
T2T_HLCOM4_invers←HLCOM	7.531***	0.818
T2T_HLCOM5_invers←HLCOM	7.900***	0.854
T2T_HLCOM6_invers←HLCOM	8.243***	0.890
T2T_HLCOM7_invers←HLCOM	5.984***	0.667
T2T_HLCOM8_invers←HLCOM	7.846***	0.853
T2T_HLCOM9_invers←HLCOM	7.235***	0.874
T2T_TRUS5←Trust	—	0.964
T2T_TRUS4←Trust	17.353***	0.924
T2T_TRUS3←Trust	9.649***	0.760
T2T_TRUS2←Trust	16.389***	0.917
T2T_TRUS1←Trust	12.453***	0.847
T2T_THEZU←HLCOM	−0.0862	−0.138
T2T_THEZU←Trust	4.033***	0.641

*Note*: Variable codes: T2, timepoint; T, Teilnahme (=participation); TRUS, scale “trust in HCP”; HL‐COM_invers, inverted scale “health literacy‐sensitive communication”; THEZU, scale “treatment confidence”.

*** *p*‐value <0.001.
